# Non sindromic cleft lip and palate: relationship between sex and clinical extension

**DOI:** 10.5935/1808-8694.20120018

**Published:** 2015-11-20

**Authors:** Daniella Reis Barbosa Martelli, Renato Assis Machado, Mário Sérgio Oliveira Swerts, Laíse Angélica Mendes Rodrigues, Sibele Nascimento de Aquino, Hercílio Martelli Júnior

**Affiliations:** a MSc. Professor of Semiology - State University of Montes Claros. PhD student - State University of Montes Claros - Unimontes; b Dentistry student. (Scientific Initiation - CNPq scholarship holder); c PhD (Full professor at Unifenas); d MSc - Graduate Program in Health Sciences - Unimontes; e MSc. Phd Student in Stomatopathology - State University of Campinas - Unicamp); f PhD. Full Professor. Graduate Program in Health Sciences - Center of Biological and Health Sciences - Unimontes

**Keywords:** cleft lip, cleft palate, female, male

## Abstract

Cleft lip and/or palate represent the most common congenital anomalies of the face.

**Aim:**

Describe the relation between non sindromic cleft lip and/or palate and sex and severity of the clef in Brazilian population.

**Methods:**

Conducted cross-sectional study, between the years 2009 and 2011, with a population of 366 patients. Data were analyzed with descriptive statistics and using the multinomial logistic regression with an interval of 95% estimated the chances of the types of cleft lip and/or palate between sex.

**Results:**

Among the 366 cases of non sindromic cleft lip and/or palate, the more frequent clefts were cleft lip and palate, followed respectively by cleft lip and cleft palate. It is noted that cleft palate were more frequent in females, while the cleft lip and palate and cleft labial predominated in males. The risk of occurrence of cleft lip in relation the cleft palate was 2.19 times in males compared to females. While the risk of cleft lip and palate in relation cleft palate was 2.78 times in males compared to females.

**Conclusion:**

This study showed that there were differences in the distribution of the non sindromic cleft lip and/or palate between male and female.

## INTRODUCTION

Non syndromic cleft lip and/or palate (NSCL/P) (OMIM # 119530) represents the most frequent congenital malformation in the head or neck region with a prevalence of approximately 1:700 live births worldwide. Its prevalence varies according to ethnicity (Africans: 0.3:1,000; Europeans: 1.3:1,000; Asians: 2.1:1,000; Native Americans: 3.6:1,000) and socioeconomic level^1^^,^^2^. In Brazil, NSCL/P prevalence ranges from 0.19 to 1.54:1,000 live births^3^, ^4^, ^5^, ^6^. NSCL/P etiology, which involves both genetic and environmental factors, is highly complex, and the molecular basis remains largely unknown^7^^,^^8^.

Affected children with NSCL/P need multidisciplinary care from birth until adulthood and have higher morbidity and mortality throughout life than do unaffected individuals^9^^,^^10^. Findings of studies have shown an increased frequency of structural brain abnormalities^11^ and that many children and their families are affected psychologically to some extent^12^.

NSCL/P can be broadly divided into those that affect the lip only, both the lip and palate, or the palate alone. Although cleft lip and cleft lip and palate are traditionally collapsed to form the single group of cleft lip with or without cleft palate, data suggest that these two categories may have different genetic causes and should, when feasible, be analysed separately^13^^,^^14^.

Cleft lip with or without cleft palate is most frequent in males, and isolated cleft palate is most typical in females, across various ethnic groups; the sex ratio varies with severity of the cleft^15^, presence of additional malformations, number of affected siblings in a family, ethnic origin, and possibly paternal age^16^^,^^17^. In white populations, the sex ratio for cleft lip with or without cleft palate is about 2:1 (male:female)^15^. In this study we examine the relation between NSCL/P and sex and severity of the clef in Brazilian population.

## METHOD

A cross-sectional study was conducted with a population of 366 patients treated at the reference Service who presented non sindromic cleft lip and/or palate, between 2009 and 2011. This reference Service of the Brazilian Health Department comprises a multidisciplinary team of health care specialists, including plastic and dental surgeons, dentists, psychologists, pediatricians, genetic counseling, nutritionists and speech therapist.

After anamnesis, physical examination was conducted to establish the kind of cleft and anatomical structures involved. The clefts were categorized in 3 parts, with the incisive foramen reference^18^: (1) *cleft lip:* include complete or incomplete clefts pre foramen, uni or bilateral; (2) *cleft lip and palate:* include uni or bilateral transforamen clefts and pre or post foramen clefts and (3) *cleft palate*: include all post foramen clefts, completes or incompletes. The patients were evaluated by professionals with large experience with oral cleft.

All patients were screened for the presence of associated anomalies or syndromes, and only those identified to have non sindromic cleft lip and/or palate were included in this study. Patients who received any surgical treatment to correct the clefts were excluded of this analysis thus carriers out of proposed classification and with cleft syndromes.

The information collected was stored in a database and analyzed using statistical program PASW® version 18.0 for windows (Chicago, USA). Data were analyzed with chi-square test (χ^2^) and using the multinomial logistic regression with an interval of 95% estimated the chances of the types of cleft lip and/ or palate between sex. This study was submitted to the Ethics Committee in Research of the University (# 129/2009). All patients or their familiars were informed about the study's purpose before they consented to participate.

## RESULTS

Among the 366 patients treated during 2009-20011, 199 (54.5%) were male and 167 (45.6%) were female. Of the total number of participants (n = 366), 25 (6.83%) presented a positive history of cleft in their families and 341 (93.16%) presented a negative history. Table 1 demonstrated the cleft distribution according to type and extension. The more frequent clefts were cleft lip and palate (53.4%), followed respectively by cleft lip (26.2%) and cleft palate (20.49%).

**Table 1:** General distribution of non syndromic cleft lip and/or palate according to type and extension.

Type of cleft	n	(%)
Cleft lip and palate	195	53.4
Cleft lip	96	26.2
Cleft palate	75	20.4
Total	366	100

In Table 2, it is possible to observe the distribution of NSCL/P according to sex.

**Table 2:** Distribution of non syndromic cleft lip and/or palate according to extension and sex.

	Cleft palate		Cleft lip		Cleft lip and palate	
Sex	n	(%)	n	(%)	n	(%)
Male	27	13.6	53	26.6	119	59.8
Female	48	28.7	43	25.7	76	45.5
Total	75		96		195	

*p:* 0.001.

There were differences between the cleft groups according to the sex. It is noted that cleft palate were more frequent in females (28.7% *versus* 13.6%; 1.77:1), while the cleft lip and palate (59.8% *versus* 45.5%; 1.56:1) and cleft labial (25.7% *versus* 26.6%; 1.23:1) predominated in males (*p* = 0.001; %^2^).

The Table 3 showed the multinomial logistic regression analysis. It turns out that the risk of occurrence of cleft lip in relation the cleft palate was 2.19 times in males compared to females. While the risk of cleft lip and palate in relation cleft palate was 2.78 times in males compared to females.

**Table 3:** Multinomial logistic regression analysis. Distribution of cleft lip and palate and cleft lip according to sex, with reference to the cleft palate.

Cleft lip	n (%)	β	OR	*p*
Sex				
Male	53 (26.6)	0.784 (0.316)	2.19 (1.18-4.07)	0.013
Female	43 (25.7)	1.00	1.00	
Cleft lip and palate				
Sex				
Male	119 (59.8)	1.024 (0.282)	2.78 (1.60-4.84)	0.000
Female	76 (45.5)	1.00	1.00	

R^2^*Nagelkerke:* 0.043.

## DISCUSSION

A recent work suggested that there has been a gross underestimation of the consequences of being born with cleft lip and/or palate^19^. Individuals born with cleft lip and/or palate, have a shorter lifespan, with increased risk for all major causes of death, when compared with individuals born without clefts^10^. Contributing to these higher mortality rates are probably psychiatric disorders and cancer. Cleft lip and/or pala te, increases the risk of hospitalization for psychiatric diseases in adults^10^. Furthermore, there is an increased occurrence of breast and brain cancer among adult females born with cleft lip and/or palate, and an increased occurrence of primary lung cancer among adult males born with cleft lip and/or palate^20^^,^^21^.

Cleft lip with or without cleft palate is listed as a feature of more than 200 specific genetic syndromes, and isolated cleft palate is recorded as a component of more than 400 such disorders^22^. The proportion of orofacial clefts associated with specific syndromes is between 5% and 7%^23^. Although the presence of a genetic component both in syndromic and nonsyndromic cleft lip and/or palate has been clearly demonstrated, only in a limited portion of a cases the genes involved have been so far identified, and in many cases, only preliminary data are available about the mechanisms leading to the craniofacial defects in the presence of a specific gene alteration^24^. In this present study, we evaluated only patients with NSCL/P. All cases with associated or syndromes were excluded.

Different studies have been conducted worldwide to evaluate NSCL/P distribution, often resulting in varying prevalence rates^10^^,^^25^. In a Brazilian population study, we showed predominance of cleft lip and palate (52.6%), followed by cleft lip (33.12%) and cleft palate (14.28%)^26^. In most published studies, the percentage of subjects with cleft lip and palate has been higher compared to that of cleft lip or cleft palate alone, including the Brazilian studies^27^^,^^28^. The findings of the present study reveal that of the 366 patients with NSCL/P, the prevalence of cleft lip and palate (53.4%) was significantly higher that of cleft lip (26.2%) and cleft palate (20.49%). Epidemiological data across different populations have shown that the prevalence of cleft palate is generally lower than that of cleft lip and palate and cleft lip and families at high risk for one type of cleft are not at increased risk for the other type, reflecting the distinct developmental origins of each form of cleft^29^. Occasionally, however, both cleft lip and palate and cleft lip and cleft palate can occur within the same pedigree, suggesting at least some overlap in the aetiology of these two broader categories of clefts^2^.

In Japanese populations, cleft lip and palate shows a significant male excess, but this excess is not seen for cleft lip alone^30^. In white populations, the male excess in cleft lip with or without cleft palate becomes more apparent with increasing severity of cleft and less apparent when more than one sibling is affected in the family^31^^,^^32^. By contrast, the male predominance in cleft lip with or without cleft palate is smaller when the infant has malformations of other systems^16^, and findings of one large study suggest predominance in females when the father is age 40 years or older^33^. Here, we showed that cleft palate were more frequent in females (28.7% *versus* 13.6%; 1.77:1), while the cleft lip and palate (59.8% *versus* 45.5%; 1.56:1) and cleft labial (25.7% *versus* 26.6%; 1.23:1) predominated in males (*p* = 0.001). It was also noted, by analyzing multinomial logistic regression that the risk of occurrence of cleft lip in relation the cleft palate was 2.19 times in males compared to females. While the risk of cleft lip and palate in relation cleft palate was 2.78 times in males compared to females. These results shall ratify the found in the literature on foreign populations^15^^,^^30^.

## CONCLUSION

This study observed a predominance of cleft lip and palate (53.4%), followed respectively by cleft lip (26.2%) and cleft palate (20.49%). There were also differences between the cleft groups according to the sex. It is noted that cleft palate were more frequent in females, while the cleft lip and palate and cleft labial predominated in males. The risk of occurrence of cleft lip in relation the cleft palate was 2.19 times in males compared to females. While the risk of cleft lip and palate in relation cleft palate was 2.78 times in males compared to females. Molecular and genetic studies are needed to better understand the differences between the types of oral clefts and sex.
